# A Variation Code Accounts for the Perceived Roughness of Coarsely Textured Surfaces

**DOI:** 10.1038/srep46699

**Published:** 2017-04-25

**Authors:** James M. Goodman, Sliman J. Bensmaia

**Affiliations:** 1 Committee on Computational Neuroscience, University of Chicago, Chicago, IL, 60637, United States; 2 Department of Organismal Biology and Anatomy, University of Chicago, Chicago, IL, 60637, United States.

## Abstract

For decades, the dominant theory of roughness coding in the somatosensory nerves posited that perceived roughness was determined by the spatial pattern of activation in one population of tactile nerve fibers, namely slowly adapting type 1 (SA1) afferents. Indeed, the perceived roughness of coarsely textured surfaces tracks the spatial variation in SA1 responses – the degree to which response strength varies across SA1 afferents. However, in a later study, the roughness of a different set of dot patterns was found to be a monotonic function of dot spacing, a result interpreted as evidence that roughness was determined by the strength of SA1 responses – the population firing rate – rather than their spatial layout. Then again, the spatial variation hypothesis was not tested directly as afferent responses to the conflicting patterns were not measured. To fill this gap, we simulated afferent responses to the dot patterns used in these roughness coding experiments using a model of skin mechanics. We then implemented the spatial variation and firing rate models of roughness based on these simulated responses to generate predictions of perceived roughness. We found that the spatial variation model accounts for perceived roughness under all tested conditions whereas the firing rate model does not.

A major question in neuroscience is how patterns of activation in sensory neurons give rise to percepts. To understand how sensory stimuli are encoded in the nervous system, it is necessary to establish not only how stimulus features are reflected in patterns of neuronal activity, but also how these activation patterns give rise to appropriate sensory percepts^1^. The study of the neural basis of tactile roughness perception has been a paradigmatic example of how to establish a neural code. First, the spatial configuration of embossed dot patterns scanned across the skin was shown to be reflected in the spatial pattern of activation evoked in slowly adapting type 1 (SA1) fibers recorded from Rhesus macaques^2^. Then, the variation in this spatial pattern – the degree to which the strength of the evoked response varies over the spatial distribution of afferents – was shown to co-vary with judgments of perceived roughness for these same surfaces measured in psychophysical experiments with human observers^3^,^4^,^5^. This neural code made intuitive sense because a perfectly flat surface excites SA1 afferents distributed across the fingertip in similar ways, resulting in low spatial variation, whereas a texture consisting of tall, skinny dots arranged in a moderately dense configuration excites some SA1 afferents (those whose receptive field falls over a dot) and not others (those whose receptive fields fall between dots). The former is perceived as smooth, the latter as rough. That spatial variation in SA1 responses accounted for roughness judgments across all tested conditions, where other neural codes failed, was taken as evidence that this aspect of the nerve activity determined perceived roughness.

This spatial code was later shown to only tell part of the story on the neural basis of roughness. Indeed, the perception of fine textural features – measured on the micron or submicron scale – was later shown to rely on temporal patterns of activation evoked in two other afferent populations^6^. However, another study questioned its validity altogether^7^. Indeed, one of the key features identified by Johnson and colleagues was an inverse U-shaped curve describing roughness as a function of dot spacing (also see Drewing, 2016)^8^: Dot patterns were roughest when dots were separated by 3 mm and decreased when the dot spacing deviated from this value; the shape of this function could be readily explained from the proposed neural code. The subsequent study found roughness judgments to be a monotonic function of dot spacing for very tall dots (height >1 mm). This pattern, the authors argued, could not be explained by the spatial variation hypothesis and rather suggested that roughness is determined by the mean firing rate evoked in SA1 afferents. Furthermore, Sutu *et al*. contended that dot patterns with small and large dot spacings evoke qualitatively different roughness percepts, which called into question the validity of the inverse U-shaped function. However, no neurophysiological data were available to demonstrate that the monotonic function of roughness vs. spatial period for tall dots could be explained by a firing rate code.

Afferent responses to spatial patterns are shaped in complex ways by skin mechanics and are therefore difficult to intuit from the stimulus. To draw definitive conclusions about neural coding in touch without analyzing the underlying neuronal representation (or at least the tissue response) is therefore impossible. In the present study, we investigated whether the spatial hypothesis of roughness perception could, in principle, account for seemingly discrepant results. To this end, we invoked a model of mechanotransduction^9^, which accurately predicts afferent responses to spatial patterns indented into the skin, to explicitly test whether the spatial variation model can account for the perceived roughness of both tall and shallow dot patterns. More generally, we assessed the extent to which the two models of roughness coding – the spatial variation model and the firing rate model – could account for roughness judgments across the range of stimulus conditions tested. First, we replicated the psychophysical results obtained by Johnson’s group based on simulated afferent responses. Second, we simulated responses to the two more recent data sets^6^,^7^ and showed that the spatial variation code can, indeed, account for these perceptual judgments of perceived roughness. Finally, we showed the firing rate model accounts for roughness judgments in some conditions but not others. We conclude that roughness perception is driven, at least in part, by the spatial variation in SA1 responses.

## Methods

### Stimuli used in source data

**Figure 1:**
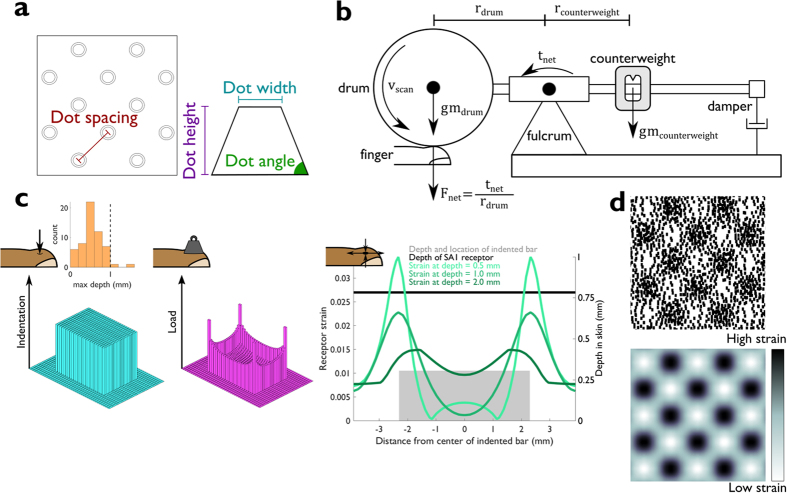
Methods. (**a**) Top view of an embossed dot pattern and side view of an individual dot. Dot patterns can be described with just four parameters. (**b**) Diagram of apparatus used to scan dot patterns across the skin, adapted from (Johnson & Lamb 1981). The drum is lowered onto the fingertip and spins with velocity v_scan_ to scan a dot pattern mounted to its circumference across the finger. The force, F_net_, with which the drum contacts the fingertip is a function of the net torque, t_net_, at the fulcrum of the lever, which is adjusted by moving a counterweight at the other end of the fulcrum. A damper ensures that sharp jolts in contact force or drum displacement are kept to a minimum. (**c**) Schematic of the skin mechanics model (adapted from Sripati *et al*.^9^). *Left:* Pattern of skin indentations. Inset depicts the distribution of maximum indentation depths produced across all textures. Note that depths are not identical to the distribution of actual heights as some dots do not indent the skin all the way, particularly when dots are relatively dense. *Middle:* Pattern of forces exerted on the skin by the pattern. Edges and corners tend to bear most of the load. *Right:* Cross section of the strains produced by the indented bar experienced by receptors at different depths in the skin. The spatial distribution of forces changes as they propagate through the tissue, which results in depth-dependent patterns of strain. (**d**) Spatial event plots (SEPs). *Top:* Spike raster of a typical SA1 afferent aligned with the locations of the dots in panel a. *Bottom:* Profile of simulated strains caused by the same dot pattern. Locations of greatest strains align with the locations of the dots in the pattern (see (**a**), *Left*). Strains are used as a proxy for SA1 firing rates.

Stimuli were Braille-like dot patterns, each consisting of an array of truncated cones arranged in a tetragonal grid. The appealing aspect of dot patterns is that they can be described using just four parameters (Fig. 1a): Dot height, dot width (the diameter of the top surface of each dot), dot spacing (the separation between the centers of two adjacent dots), and dot angle (the angle between the lateral surface of a dot and the flat base from which it is embossed). In the first set of experiments by Johnson and colleagues (dataset A), dot width ranged from 500 to 1200 microns and dot spacing from 1.3 to 6.2 mm with dot height constant at 370 microns^4^. In the second (dataset B), dot width ranged from 250 to 2500 microns, dot height from 270 to 620 microns, and the spacing was constant at 3.5 mm^3^. In the third (dataset C), dot spacing varied from 2 to 6 mm, with a constant dot width of 500 microns and dot height of 740 microns^6^. In the data collected by Chapman’s group (dataset D), dot height varied over a wide range (from 360 to 1800 microns)^7^, with a constant dot width of 700 microns, dot angles that co-varied with dot height to maintain a constant base diameter of 1400 microns, and dot spacings that ranged from 1.3 to 6.2 mm (cf. Connor *et al*.^4^). See Table 1 for summary of each dataset. Here, we simulate the responses of populations of SA1 afferents to these various stimuli to replicate and extend previous results.

### Psychophysics methods for source data

The objective of the present study was to determine whether the spatial variation and firing rate hypotheses could account for psychophysical judgments of roughness from the aforementioned studies (dataset A–D). Briefly, human subjects pressed their finger lightly onto a rotating cylindrical drum or had the rotating drum lowered onto their finger. The surface of the drum was embossed with a regular array of Braille-like dots. The cylinder was balanced with a counterweight so that a constant force was exerted on the finger pad (see Fig. 1b for a schematic). Subjects were instructed to produce magnitude estimates of the perceived roughness of each surface on a numerical scale. Subjects were further instructed to treat the estimates as a proportional measure of roughness; that is, if one texture felt twice as rough as another, it was to be ascribed a number that was twice as large. Each subject was presented with a sequence of stimuli that spanned the range of parameters. Ratings were first normalized by dividing by the grand mean of each subject’s ratings and then pooled across subjects. All psychophysical data in the present study are from past publications. These methods were carried out in accordance with relevant guidelines and regulations and were approved by the Institutional Review Boards (IRB) of Johns Hopkins University (datasets A and B), of the University of Chicago (dataset C), and the institutional ethics committee of the University of Montreal (dataset D). All subjects participating in these studies provided informed consent.

### Neurophysiology for source data

We validated our simulation by comparing simulated responses to their measured counterparts from previous work^3^,^4^. Neurophysiological methods are described at length in these previous studies and are only summarized here. Individual cutaneous nerve fibers were dissected from the median and ulnar nerves of anesthetized Rhesus macaques and wrapped around a silver electrode. After localizing the receptive field (RF), the afferent class was identified using standard methods: SA1 fibers are the only ones to both have small receptive fields and to respond with a sustained response to a ramp-and-hold indentation of the skin. The rotating drum was then lowered onto the fingerpad and the dot patterns were scanned across the finger while afferent responses were recorded. To reconstruct the activity evoked in a population of identical afferents with RFs that tile the skin, textures were scanned across the skin, then the cylinder was translated by 200 microns along the axis perpendicular to the scanning dimension, and the textures were re-scanned. This procedure was repeated until the entire surface of the stimulus cylinder had been scanned across the fiber’s RF. Spike rasters were then replotted to construct spatial event plots (SEPs), which align the timing of each spike to the location of the stimulus at which it occurred. An SEP is a reconstruction of the neural image of the surface carried by populations of SA1 afferents (Fig. 1d). As with the psychophysical data, all neurophysiological data in the present study are from past publications. All procedures to collect these data followed all relevant guidelines and were approved by the Institutional Animal Care and Use Committees (IACUC) of Johns Hopkins University (datasets A and B) and the University of Chicago (dataset C).

### Skin mechanics and simulated afferent responses

When a stimulus is indented into the skin, forces propagate through the tissue and ultimately deform mechanoreceptors embedded into the skin, a process that is well approximated using continuum mechanics^9^,^10^. In these models, the skin is assumed to be incompressible, isotropic, elastic, and infinite in scale from the perspective of each mechanoreceptor. Here, we first expressed each stimulus as an array of skin indentations with a density of 25 mm^−2^ (that is, with 200 micron separation between adjacent pixels). We then calculated the loads exerted on the skin’s surface using point load mechanics^11^:


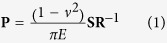


where **P** is a 1 × *n* vector of loads exerted at each point of the array, with *n* being the number of points in the array; **S** is a 1 × *n* vector of stimulus indentation heights at each point of the array; **R** is a *n* × *n* matrix of distances between pairs of points on the array (a symmetric matrix where r(*X, Y*) = r(*Y, X*) is the distance between points *X* and *Y* on the array); *E* is the Young’s modulus of the skin in response to strains normal to the surface; and *ν* is the Poisson’s ratio of the skin. The value of *E* was assumed to be 50 kPa for human skin, and *ν* was assumed to be 0.4.

In brief, equation (1) states that a load experienced at some location X by an indentation at location Y is proportional to the depth of the indentation at point Y and inversely proportional to the distance between X and Y. In general, the edges of indented patterns tended to be enhanced in the load distributions while internal features of the patterns tend to be obscured (Fig. 1c). The initial force computation assumes that every probe exerts a force on the skin, contributing to the overall displacement. However, a large indentation into the skin will prevent a smaller indentation from contacting the skin if the latter is sufficiently near the former. When the small indentation is included in the force computation, a negative load is assigned to that stimulus component to force the skin to conform to the stimulus, implying incorrectly that this stimulus component pulls on the skin. To correct for this, we removed stimulus components to which negative loads were assigned from the force computations. Because the removal of stimulus components causes a redistribution of the forces exerted by the remaining stimulus, we recomputed the forces after excluding components exerting negative loads and repeated these steps until there were no negative values in the load vector P.

We then estimated the propagation of these forces through the skin to estimate the stresses and strains generated on Merkel cells, the mechanoreceptors that drive SA1 responses^9^. The propagation of forces through the tissue tends to distort the spatial pattern presented to the surface (Fig. 1c). To reconstruct the activation of populations of SA1 afferents, we computed strains at a depth of 0.77 mm to simulate SA1 afferent receptor depths. The maximum compressive strain was taken to be the strain component that drives SA1 afferent spiking, as this quantity has been found to be linearly related to SA1 firing rate^9^ though most other strain and stress components yield virtually identical predictions. Strains were then used as a proxy for neuronal firing.

In psychophysical experiments with dot patterns, stimuli were delivered such that they exerted a specific amount of force on the skin. We simulated the fixed force protocol by iteratively increasing the indentation of the stimulus into the skin until the overall force matched that in the corresponding experiment. The iterative process to equate contact force had little effect on shallow dot patterns, for which the fingertip is in contact with the entire stimulus surface. However, for tall dot patterns from dataset D, adjusting contact force affects how much of the surface the skin contacts, which in turn affects the evoked neuronal response. In addition, although the tall dots reached a maximum height of 1.8 mm (beyond the range of indentation depths for which the skin mechanics model has been validated), the fixed-force protocol actually raised these dot patterns relative to the skin, thereby reducing the maximum indentation imposed by these dot patterns. Figure 1c (left inset) shows the distribution of maximum depths of indentation imposed by all simulated textures in this study. Only two of the 1.8 mm-tall textures from dataset D achieve a maximum indentation depth greater than 1 mm, with both of these textures having the two largest dot spacings from that dataset. Even for these dot patterns, no more than 2% of probes exceeded a depth of indentation into the skin of 1 mm during simulated scans across the fingertip. Therefore errors in the strain estimates of the skin mechanical model for large skin deflections are unlikely to have had a meaningful effect on roughness predictions (see below).

### Roughness models

We implemented two different models of how the pattern of skin strains estimated by the skin mechanics model, and therefore the pattern of simulated SA1 afferent activation, drives perceived roughness. According to the first model^7^, roughness is determined by the mean firing rate across the population of SA1 afferents, estimated by taking the mean strain at the depth of Merkel cells (a proxy for mean firing rate). According to the second model, roughness is determined by the spatial variation in SA1 responses^5^. We computed spatial variation from the strain profile using an algorithm and parameters that were identical to those described previously^3^,^4^,^5^. Briefly, 2D Gabor filters varying in orientation and phase were convolved with the spatial pattern of strains generated by each dot pattern. The absolute value of the convolution was then summed over the stimulus and across filters to obtain a measure of spatial variation in the response evoked by each textured surface. This measure of spatial variation, which is highly predictive of roughness judgments in the Johnson studies, implies a computation of the difference in activity of mechanoreceptors separated by a given distance (determined by the period of the sinusoid). More general measures of spatial variation, for example the variance in response across afferents, have been found to be less predictive of perceived roughness^3^,^4^,^5^. An appealing aspect of using Gabor filters to describe the spatial variation computation is that these filters provide an accurate description of the receptive fields of cortical neurons^12^ (see below). Note that filter parameters were set to previously reported values, so no optimization was necessary to compute spatial variation.

According to Johnson^1^, a neural code should be linearly predictive of its associated perceptual dimension. With this in mind, we computed the linear correlation between mean firing rate or spatial variation and perceived roughness (and derived the coefficient of determination, *R*^*2*^) for each data set and used it as the fitness measure to compare the two candidate neural codes.

## Results

### Simulated SA1 responses to embossed dot patterns

**Figure 2:**
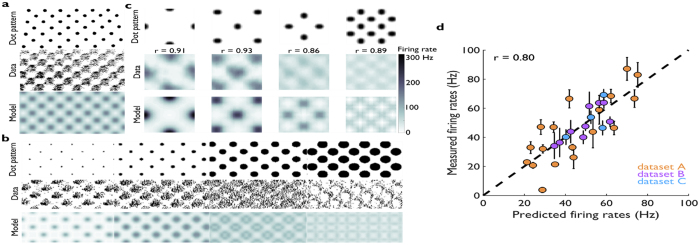
Measured and reconstructed neuronal responses. (**a**) Top: Example dot pattern for which measured SA1 responses were available. Middle: SEP constructed from measured responses. Bottom: SEP constructed from simulated strains (adapted from Connor *et al*.^4^). (**b**) Top: Four dot patterns used in a separate experiment. Middle: Corresponding SEPs constructed from measured neural responses to these patterns. Bottom: SEPs constructed from simulated strains. Both sets of SEPs transition from faithful snapshots of dots (left) to “holes” in the pattern of spiking at dot locations (right) (adapted from Blake *et al*.^3^). (**c**) Top: Dot patterns; Middle: SEPs constructed from smoothed measured responses; Bottom: SEPs constructed from simulated strains. Pixel-by-pixel correlation coefficients between model and measurement are shown above each pair of SEPs. (**d**) Measured vs. predicted overall mean firing rates of SA1 afferents in response to each texture from datasets A^4^, B^3^ and C^6^. Error bars depict standard error of the mean for measured rates.

The patterns of strains generated by the model match the SEPs from datasets A–C, for which neurophysiological responses were collected^3^,^4^,^6^. For these stimuli, the spatial pattern of SA1 activation is a blurred version of the grid of dots (Fig. 2a,c), as are the strain profiles. Furthermore, patterns from dataset B, comprising large-diameter dots, evoke SEPs in which most of the spikes are evoked at the edges of the dots and few spikes are evoked over most of the dots’ surfaces^3^. Again, model predictions match this behavior (Fig. 2b), as the centers of the dots are associated with low strains. The model not only replicates the spatial patterning in the measured responses but also their strengths. The mean strain evoked by each dot pattern is proportional to the mean SA1 firing rate that is evoked by the same dot pattern (Fig. 2d). The model thus provides an accurate reconstruction of SA1 responses to embossed dot patterns, as it does of the other spatial patterns on which it has been tested^9^.

### Predicting roughness using rate-based and spatial variation codes

**Figure 3:**
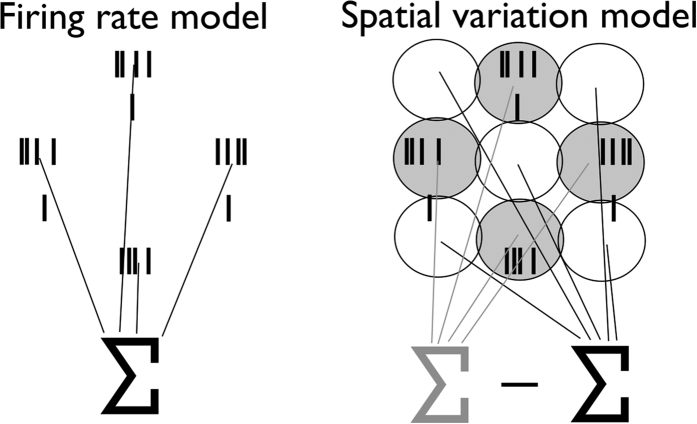
Schematic of roughness models. Left: According to the firing rate model, all spikes, regardless of spatial location, are summed to generate a roughness percept. Right: According to the spatial variation model, the differences in spike counts evoked in spatially displaced SA1 afferents (at a distance of approximately 2 mm) determines roughness.

**Figure 4:**
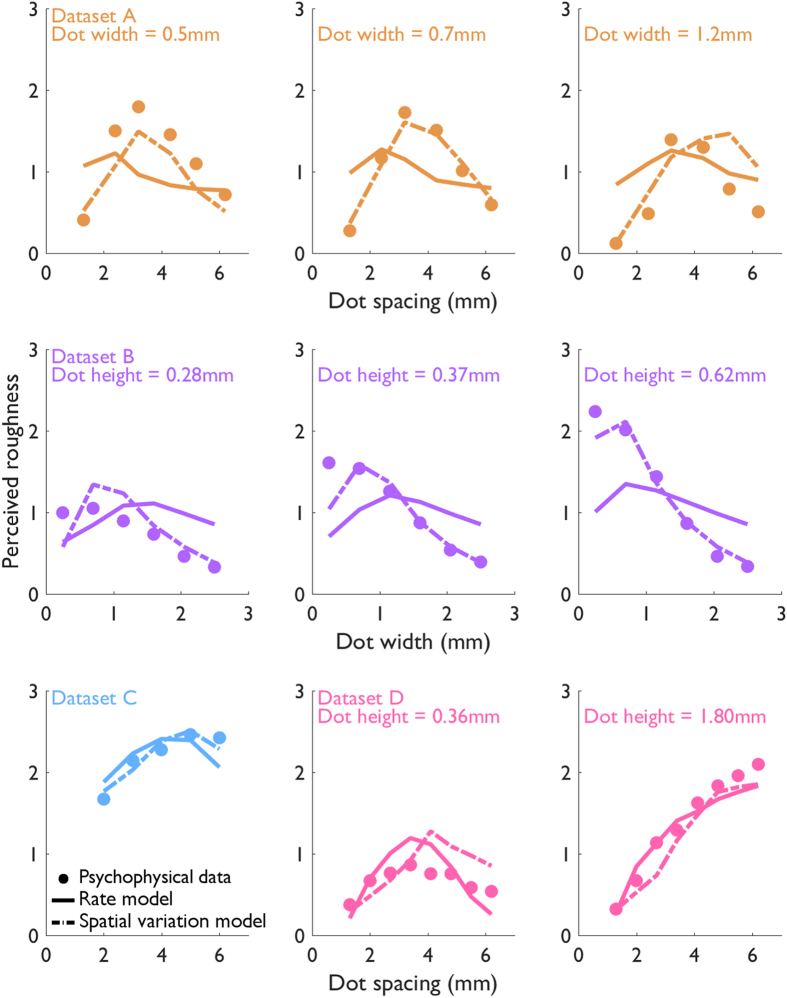
Measured and predicted roughness. Normalized roughness judgments obtained in psychophysical experiments (circles) along with roughness predicted by the mean rate (solid lines) and spatial variation (dashed lines) models. Orange: dataset A^4^; Purple: dataset B^3^; Blue: dataset C^6^; Pink: dataset D^7^.

The goal of the present study was to examine the degree to which firing rate and spatial variation models (diagrammed in Fig. 3) could account for the perceived roughness of dot patterns. We found that, while the firing rate code accounts for the perceived roughness of dot patterns from dataset D, this code could not account for the perceived roughness of shallower dots from datasets A–C (Fig. 4). Indeed, while roughness is strongly modulated by dot spacing and dot width for these patterns, rate modulation is weak and does not match its psychophysical counterpart. The poor predictions of the rate-based model replicate findings by Johnson and colleagues for these stimuli, who concluded that rates could not account for perceived roughness^4^,^5^.

**Figure 5:**
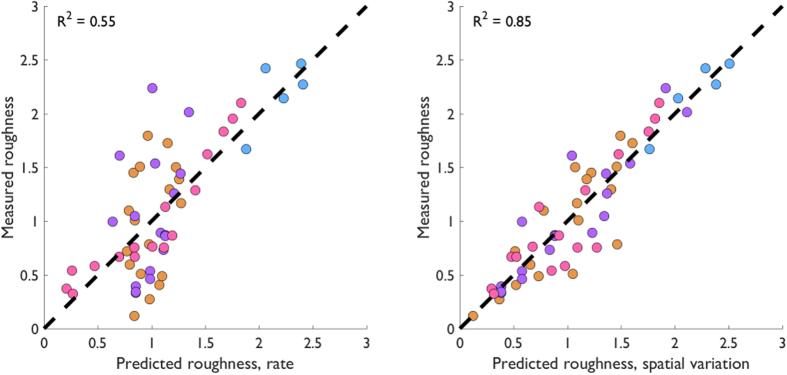
Firing rate and spatial variation model predictions. Measured vs. predicted roughness for the firing rate model (left) and spatial variation model (right). The spatial variation model provides more accurate predictions than does the firing rate model across all conditions tested. Colors indicate separate datasets using the same conventions as in Figs 2 and 4.

In contrast, the spatial variation code accounts for perceived roughness across all tested conditions. Overall, the spatial variation model accounts for 85% of the variance in roughness judgments whereas the firing rate model accounts for 55% of the variance (Fig. 5). The difference between these two measures of linear fit is highly statistically significant (Williams-Hotelling test: *t(45*) = −4.50, *p* = 4.80e-5). On the other hand, the firing rate model accounts for more variance (87%) in dataset D than does the spatial variation model (80%), but this difference is not significant (Williams-Hotelling test: *t(13*) = 0.88, *p* = 0.393). Furthermore, roughness judgments from datasets A and B, when considered in isolation rather than pooled, were better accounted for by the spatial model than by the firing rate model. A summary of the model performance statistics for each model based on simulated responses is provided in Table 2.

**Figure 6:**
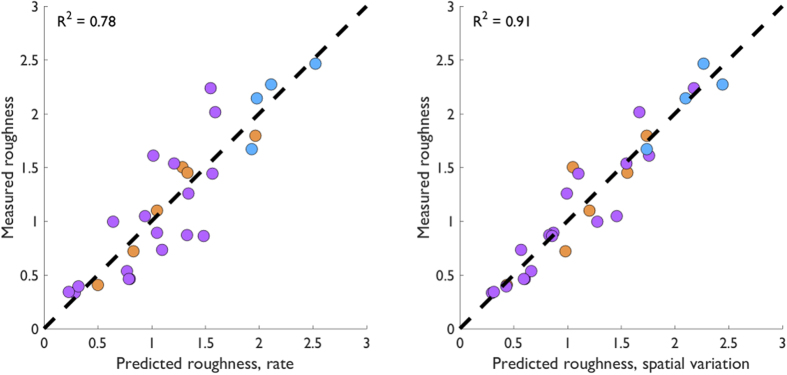
Roughness predictions using measured afferent responses. Measured vs. predicted roughness for the firing rate model (left) and spatial variation model (right) applied to the neural spiking data shown in Fig. 2. Colors indicate separate datasets using the same conventions as in Figs 2, 4 and 5.

To verify that our results were not an artifact of our implementation of the skin mechanics model, we also calculated roughness estimates from measured responses to the subset of dot patterns for which those data were available (Fig. 6)(shown in Fig. 2). Within this subset of data, we still found that the spatial variation model accounts for more variance in the roughness estimates (91%) than does the firing rate model (78%)(Williams-Hotelling test: *t(22*) = −2.33, *p* = 0.03). A summary of the model performance statistics based on neurophysiological data is provided in Table 3.

To account for the two dot patterns from dataset D that were sparsely populated by pins indenting the skin beyond 1 mm, we used an alternate skin mechanics model wherein strains resulting from indentation depths beyond the first 1 mm would be subject to an elastic modulus multiplied one-thousand fold. This roughly approximates the higher resistance of skin to further indentation beyond the first 1 mm^13^. As expected, the predictions of the modified model are very similar to those of a model that assumes a constant modulus of skin (Table 4) and yields similar conclusions regarding roughness codes: the firing rate and spatial variation models account for 88% and 81% of the variance in the roughness estimates from dataset D, respectively, a difference that is not statistically significant (Williams-Hotelling test: *t(13)* = 0.89, *p* = 0.391).

## Discussion

### The spatial variation code accounts for perceived roughness

Our modeling results demonstrate that a spatial variation code can, in principle, account for the perceived roughness of all dot patterns for which psychophysical data have been published, including those created by Chapman’s group^7^. Indeed, spatial variation in simulated neuronal responses accounts for 85% of roughness judgments across all experiments without any systematic deviations between predicted and measured data. In this view, roughness perception does not involve simply integrating the strength of the response across nerve fibers but rather entails a computation of variation applied to the spatial pattern of activation over the receptor sheet, a true population code.

Note that an appealing aspect of the spatial variation hypothesis is that the spatial layout of the afferent response is less sensitive to changes in scanning speed than is the response rate. Thus, not only does spatial variation account for the perceived roughness when textures are scanned at a given speed, but it can also account for the documented constancy of roughness perception across speeds^14^,^15^,^16^,^17^.

### Model performance and assumptions

In the present study, we use estimated strains at the location of Merkel receptors in the skin as a proxy for SA1 afferent firing rate. These strains have been shown to be linearly predictive of SA1 firing rates in previous work, accounting for 75% of the variance in afferent responses to a broad range of stimuli, including square-wave gratings, spheres, oriented bars, single probes, and annuli^9^. The ability of the model to predict roughness of scanned dot patterns across a wide range of conditions is surprising because it does not take into consideration (1) tangential forces applied at the surface of the skin and (2) any spiking related dynamics, which have been shown to shape afferent responses to dynamic stimuli^18^,^19^,^20^. Lack of tangential forces excludes any contribution to roughness coding from slowly adapting type 2 fibers, which had previously been implicated in roughness perception^21^. However, the paucity of SA2 fibers in the glabrous skin^22^ undermines this hypothesis. Furthermore, tangential and normal forces are highly correlated during texture scanning^23^, so including the former is likely to have little impact on the results. The spiking mechanism plays a critical role in shaping the precise spike timing of mechanoreceptive afferents^24^, but the spatial patterns at the resolution at which they are analyzed here are relatively insensitive to precise spike timing at the precision these spiking models afford. Despite the exclusion of tangential forces and of spiking mechanisms, the reconstructed neuronal responses matched their measured counterparts in both strength and spatial patterning, even though dot patterns were not used to develop the model in the first place. Given the remarkable fit to measured responses to dot patterns, it is likely that the reconstructed responses to dot patterns for which neurophysiological data are not available (dataset D) are comparable in fidelity.

One might argue that the lack of fit of the firing rate model might be attributable to the use of strain as a proxy for firing rate. However, we show that the firing rate model accounts well for the roughness of the divergent patterns (dataset D) but fails for the other datasets, which is a replication of previous findings with measured neuronal responses^4^,^5^ (replotted in Fig. 6). The important result is that spatial variation *does* account for these divergent patterns.

### Spatial variation computation in somatosensory cortex

An appealing aspect of the spatial variation hypothesis of roughness coding is that a large population of neurons in primary somatosensory cortex (S1) implement a computation of such variation. Indeed, the RFs of many S1 neurons comprise an excitatory field flanked by one or more inhibitory fields, precisely the RF structure required to compute variation from a spatial pattern of input^25^. In fact, the RFs of these neurons are well approximated by Gabor functions^12^. The observation of a spatial variation computation in cortex bolsters the case that it mediates perceived roughness.

### The contribution of a temporal code to perceived roughness

Although a spatial code accounts for the roughness of dot patterns, this neural code cannot account for the roughness of surfaces that include fine features. Indeed, SA1 afferents barely respond to fine textural features and, when they do, their responses are rather uninformative^6^. To discern fine surface features requires movement between skin and surface^26^, which leads to the production of texture-specific skin vibrations that propagate across vast swaths of skin^27^,^28^,^29^,^30^,^31^. These skin vibrations, in turn, produce temporal spiking patterns in two other populations of afferents, namely rapidly adapting (RA) and Pacinian (PC) fibers. These spatially distributed temporal spiking patterns are highly informative of texture identity, as evidenced by high texture discrimination performance even following the elimination of SA1 responses in the fingertip through digital anesthesia^31^. Moreover, these texture-specific temporal spiking patterns can also account for the perceived roughness of fine textures with features too small to be resolved in the spatial pattern of activation of SA1 afferents. Specifically, the *temporal* variation in RA and PC responses – the degree to which their response strengths vary over time – determines the perceived roughness of fine textures. As most textures comprise both coarse and fine features, these two mechanisms – spatial and temporal – cooperate to shape perceived texture. In fact, the perceived roughness of surfaces that span the range of tangible textures can be predicted based on a linear combination of spatial variation in SA1 fibers and temporal variation in RA and PC fibers^6^. Nonetheless, the representation of dot patterns is dominated by SA1 responses, as evidenced by the fact that their perceived roughness can be predicted very accurately from the responses of this afferent population at the exclusion of the others.

## Additional Information

**How to cite this article**: Goodman, J. M. and Bensmaia, S. J. A Variation Code Accounts for the Perceived Roughness of Coarsely Textured Surfaces. *Sci. Rep.* **7**, 46699; doi: 10.1038/srep46699 (2017).

**Publisher's note:** Springer Nature remains neutral with regard to jurisdictional claims in published maps and institutional affiliations.

## Figures and Tables

**Table 1:** Source papers for each dataset along with a summary of experimental conditions.

Dataset	Authors	Year	Dot Heights (μm)	Dot Widths (μm)	Dot Spacings (mm)	Human psychophysics	Monkey neurophysiology
A	Connor *et al*.^4^	1990	350	500, 700, 1200	1.3, 2.4, 3.2, 4.3, 5.2, 6.2	Yes	Yes
B	Blake *et al*.^3^	1997	280, 370, 620	250, 700, 1150, 1600, 2050, 2500	3.5	Yes	Yes
C	Weber *et al*.^6^	2013	740	500	2.0, 3.0, 4.0, 5.0, 6.0	Yes	Yes
D	Sutu *et al*.^7^	2013	360, 1800	700	1.3, 2.0, 2.7, 3.4, 4.1, 4.8, 5.5, 6.2	Yes	No

**Table 2:** Summary of model performance based on simulated responses. d.o.f = degrees of freedom; W-H test = Williams-Hotelling test.

Dataset	% variance in roughness explained by mean rate	% variance in roughness explained by spatial variation	d.o.f.	T statistic (W-H test of correlations)	P value
A	10.8%	69.4%	15	−2.86	1.19e-02
B	10.6%	84.4%	15	−6.77	6.25e-06
C	49.8%	86.4%	2	−1.47	2.79e-01
D	87.5%	79.9%	13	+0.88	3.93e-01
Pooled	54.9%	84.9%	45	−4.50	4.80e-05

**Table 3:** Summary of model performance based on measured responses for a subset of data (Fig. 2).

Dataset	% variance in roughness explained by mean rate	% variance in roughness explained by spatial variation	d.o.f.	T statistic (W-H test of correlations)	P value
A	91.7%	78.2%	4	+1.05	3.52e-01
B	59.0%	88.3%	16	−2.53	2.21e-02
C	63.7%	78.7%	2	−0.32	7.81e-01
Pooled	78.0%	91.1%	22	−2.33	2.96e-02

**Table 4:** Summary of model performance for dataset D when using the unaltered (normal) model and when increasing the elastic modulus of skin 1000-fold for indentations beyond 1mm (modified).

Model	% variance in roughness explained by mean rate	% variance in roughness explained by spatial variation	d.o.f.	T statistic (W-H test of correlations)	P value
Normal	87.5%	79.9%	13	+0.88	3.93e-01
Modified	88.1%	80.9%	13	+0.89	3.91e-01
